# Salivary cortisol levels and stress in adults with profound intellectual and multiple disabilities participating in the Structured Water Dance Intervention: a randomised controlled crossover trial

**DOI:** 10.1038/s41598-022-21573-x

**Published:** 2022-10-19

**Authors:** Lars-Olov Lundqvist, Marie Matérne, Andre Frank, Evalotte Mörelius, Anna Duberg

**Affiliations:** 1 University Health Care Research Center, Faculty of Medicine and Health, Örebro University, 70182 Örebro, Sweden; 2 School of Law, Psychology and Social Work, Örebro University, Örebro, Sweden; 3 Division of Nursing Sciences and Reproductive Health, Department of Health, Medicine and Caring Sciences, Linköping University, Linköping, Sweden; 4 School of Nursing and Midwifery, Edith Cowan University, Joondalup, WA Australia

**Keywords:** Human behaviour, Rehabilitation, Randomized controlled trials

## Abstract

The Structured Water Dance Intervention (SWAN) is a dance-oriented aquatic group activity directed to give opportunities for the joy of movement, relaxation, and reduced stress. This study aimed to evaluate the effects of SWAN on salivary cortisol and stress in adults with profound intellectual and multiple disabilities (PIMD). A total of 34 adults with PIMD at four habilitation centres in Sweden completed the SWAN intervention. The intervention was administered for 40 min once a week during a 12-week period. Saliva cortisol was collected in the morning and evening at baseline one week before the intervention, thrice during the intervention period, and one week after the intervention. Moreover, in connection with the SWAN sessions, the participants’ level of stress was also assessed by the accompanying assistants. The results showed that salivary cortisol and participants stress decreased significantly, directly after the SWAN sessions compared with measures directly before sessions. The study demonstrates that adults with PIMD have diurnal salivary cortisol patterns consistent with those observed in adults without disability and that the SWAN reduces salivary cortisol levels and stress in people with PIMD; this justifies that SWAN could be considered in the choice of interventions to reduce stress in adults with PIMD.

**Trial registration:** This study is registered 09/04/2019 on ClinicalTrials.gov (ID: NCT03908801).

## Introduction

Adults with profound intellectual and multiple disabilities (PIMD) have a combination of profound intellectual disability and physical impairments with a high level of multimorbidity, often with the addition of sensory deficits and difficulty in communicating, leading to an increased need for assistance with activities of daily living^1,2^. The prevalence of PIMD is low and available data from northern Europe indicates that there are about 60 to 80 people per 100,000 inhabitants^2,3^.

It has been hypothesised that people with disability may report higher than average stress levels because of additional challenges in life^4^, and that those with neurological disorders have deficits in the integration of sensory information and increased emotional reactivity that may induce hyperarousal of the stress system^5^. In addition, people with severe intellectual disability and sensory deprivation are assumed to be more prone to experiencing stress in novel, unpredictable and uncontrollable situations because they may have insufficient resources to deal with stressors on their own^6,7^. However, research on stress and its effects on people with PIMD is greatly lacking.

Due to their profound disability, it is not possible to ask people with PIMD of their perceived level of stress, therefore, proxies are commonly used as raters. However, a frequently used objective marker of stress is saliva cortisol^8,9^. In the presence of a stressor, the hypothalamic–pituitary–adrenal (HPA) axis is activated. This gives rise to a hormonal chain response that subsequently leads to the release of cortisol into the bloodstream, with a peak in saliva approximately 25–30 min later^8,10^. Cortisol has widespread effects on physiological processes that prepare for and support coping with the stressor^11^. In addition, and under normal conditions, cortisol secretion develops a circadian diurnal pattern with high levels in the morning, a peak 30–45 min after awakening, and a slow decline during the reminder of the day with its lowest levels at night^8,12,13^.

To the best of our knowledge, there are no published studies on cortisol levels and stress related to adults with PIMD, yet. There are, however, indications that some diagnoses present in PIMD may be associated with deviating HPA axis function, such as Rett syndrome, cerebral palsy and neurological complications, at least in their more severe forms. That is, adults with Rett syndrome are shown to have a less steep diurnal saliva cortisol decline throughout the day compared with typically developing individuals^14^. Children with cerebral palsy have higher basal morning (between 9 and 12 a.m.) saliva cortisol levels immediately before attending a physical therapy session than typically developing children^15^. Research on young adults born premature with neurological complications, such as intraventricular haemorrhage or hydrocephalus, show higher saliva cortisol reactivity to social evaluative threats than adults born full-term or preterm without complications^16^. However, other studies report similar diurnal saliva cortisol patterns in adults with intellectual disabilities as compared to those found in typically developing individuals^17^. Thus, further research on saliva cortisol in people with PIMD is warranted.

As indicated by the research presented above it may be hypothesized that people with PIMD are at risk of experiencing stress. Accordingly, health care providers offer a broad range of services in order to meet their specific and often complex medical and rehabilitation needs^18^. To alleviate their situation and give them a more meaningful life, people with PIMD are offered various forms of activities and therapies, such as multiple sensory stimulation therapies in the form of massage, music, or warm water pool activities^19^. A relatively common activity available to people with PIMD in Sweden is hydrotherapy that uses the physical properties of water, such as temperature and pressure, for therapeutic purposes^20^ and may be particularly suitable for people with movement restrictions that limit their participation in land-based activities^21^.

Although, studies on the effects of hydrotherapy on cortisol levels in people with PIMD is lacking, a recent systematic review concluded that hydrotherapy has the potential to reduce cortisol levels in healthy individuals and considered it a feasible method to reduce stress^22^. In addition, staff working closely with adults with PIMD have reported aquatic interventions to be beneficial, particularly if the programme is individually tailored^23^. Thus, based on the beneficial effects of hydrotherapy and its potential to reduce stress in people with PIMD, the Structured Water Dance Intervention (SWAN) was developed.

The SWAN is a dance-oriented group activity performed in a warm pool accompanied by music to enhance the joy of movement, social interaction and to support relaxation^24^. Previous research shows that SWAN serves meaningful goals in body functions as well as in activity and participation, related to a health promoting activity for adults with PIMD^25^. Furthermore, caregivers, health personnel and disability health care managers engaged in the SWAN intervention believe that it is a suitable form of activity for people with PIMD^26^.

### Aim

The aim of the present study is to describe diurnal cortisol levels and evaluate the effects of SWAN on salivary cortisol and stress in adults with PIMD. We predict that people with PIMD have the same diurnal cortisol levels as typically developing individuals, that salivary cortisol and by proxy ratings will decrease in response to the SWAN intervention indicating no stress from the intervention and, at the end of the program, salivary cortisol evening values will be lower in SWAN group compared to the control group indicating long-term beneficial effects of the SWAN intervention.

## Methods

### Design

**Figure 1:**
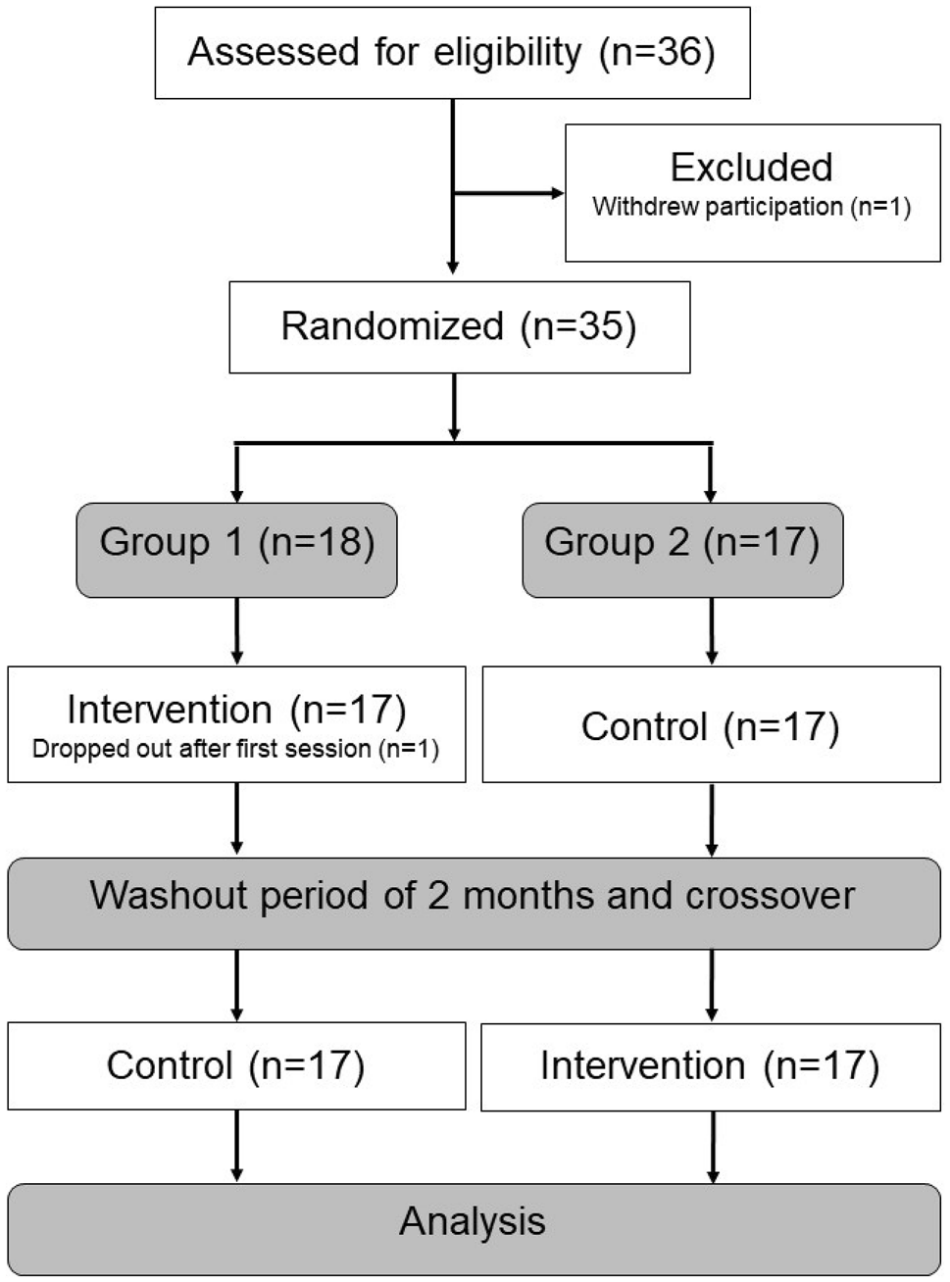
CONSORT flow chart.

This study is part of the research project SWAN, a multicentre randomised intervention study, including participants from different habilitation centres in four Swedish regions. Further details of the study design were previously reported in a study protocol^24^. At each centre there were two groups: an early intervention group (Group 1) and a late intervention group (Group 2). The participants were randomised into Group 1 or 2 by minimisation^27^ using the MINIM software^28^. Group 1 received SWAN when they entered into the study; Group 2 acted as a control group. After a washout period of two months, Group 2 received the same intervention as Group 1, while Group 1 returned to their normal activities. Consequently, all participants completed both intervention and control conditions. For a CONSORT flow chart see Fig. 1.

### Participants

Participants were recruited from habilitation centres in four regions in central Sweden by physiotherapists in collaboration with the research team, according to the inclusion criteria: being adult (18–65 years) with PIMD and having previous experience of hydrotherapy or other aquatic activities without any discomfort. PIMD was defined as having a motor impairment corresponding to Gross Motor Function Classification System level IV or V^29^ and a profound intellectual disability according to the International Classification of Diseases, tenth revision of the World Health Organization^30^. The definition was communicated with the local physiotherapists and when needed, the medical records were checked for clarification. Criteria for exclusion were: severe hearing impairment or deafness (since music was played to enhance the water dance activity) and infections or ulcers, because it could be infectious in the pool. Cerebral palsy and epilepsy were the most common diagnosis among the participants. These diagnoses were often present simultaneously and for some participants in combination with other diagnosis, such as autism spectrum disorder, other brain disorders, rare diseases, asthma/allergy, or visual impairments.

A pre study power analysis using an online service (www.stat.ubc.ca) indicated that 40 participants were needed to achieve a study power of 80% at an alpha of 5%. However, it was possible to recruit only thirty-six individuals. Two of them withdrew their participation at the beginning of the study; one withdrew participation before the first session and the other after the first SWAN session due to stomach problems and issues with tolerating group activities. Thus, 34 individuals completed the intervention, which included 20 men and 14 women. Characteristics of the study participants are shown in Table 1.

**Table 1:** Characteristics of the study participants.

Variable		
Age, M (range) (in years)	33.8	(21–53)
**Gender, frequency (%)**		
Men	20	(59%)
Women	14	(41%)
**Diagnosis; frequency (%)**^a^		
Intellectual disability	34	(100%)
Epilepsy (G40)	29	(85%)
Cerebral palsy (G80.9)	24	(71%)
Other brain disorders (G93)	3	(9%)
Autism spectrum disorders (F84)	3	(9%)
Rare diseases (Rett, Kabuki)	3	(9%)
Asthma/allergy (J45)	3	(9%)
Visual impairment (H54)	2	(6%)
**Sensitivity to stress**^b^		
Never	4	(12%)
Seldom	12	(36%)
Sometimes	10	(30%)
Often	4	(12%)
Always	3	(9%)

Most participants have more than one diagnosis; thus, the sum of percentage is larger than 100.

^a^ICD-10 code in parenthesis.

^b^Missing values on one participant.

### The Structured Water Dance Intervention

The SWAN intervention is an aquatic group activity with four to five participants in a warm pool (33–35 °C). The 40-min sessions were held around noon once a week for 12 weeks. Two of the four SWAN groups were scheduled in the morning (approximately at 11 a.m.) and the other two groups were scheduled in the afternoon (approximately at 1 p.m.). Each session followed a structured program focusing on the following key components: experience of dance and music, adapted movements, stimulation of the senses, and social interaction. A playlist of nine music tracks was created to stimulate the rhythm of the movements performed, to support relaxation, and to elicit different emotions. Two assistants accompanied each participant; one of them was in the pool and served as a dance partner while the other remained at the poolside to support when the participants entered or left the pool. The second assistant also helped out with floating devices and other practical matters. The sessions were led by two instructors. One of them instructed the group from the poolside and the other was in the water to guide the participants and the assistants with the dance movements.

### Outcome measures

#### Saliva sampling

Twenty-six samples were planned to be collected for each participant, 16 samples during the intervention condition and 10 samples during the control condition. The time schedule for saliva sample collection is shown in Table 2.

**Table 2:** Time schedule for saliva sample collection.

	1 week before	Session 1	Session 6	Session 12	1 week after
**Control**					
Morning	X	X	X	X	X
Evening	X	X	X	X	X
**Intervention**					
Morning	X	X	X	X	X
Before entering the pool		X	X	X	
Before exiting the pool		X	X	X	
Evening	X	X	X	X	X

Saliva was collected morning and evening at the participants’ home by the caregivers and at the pool in connection with the intervention by a physiotherapist in the research group. At all sites, salivary cortisol was collected using a sampling equipment (Salimetrics LLC, State College PA) comprising a swab (SalviaBio Children’s Swab) and a centrifugation tube.

For the salivary cortisol collections at the participants’ home, sample kits were arranged and complemented with disposable gloves for hygiene reasons. All tubes were prelabelled with one number specific for each participant and one number representing the occasion the sample was planned to be collected. The sampling kits were distributed to the participants’ home with a written step by step instruction and a diary to record sampling information (date and time), and contact information for questions. Additional instructions were given if needed with a short information video clip by Salimetrics on how to perform the saliva sampling. Moreover, an information video on how to record/handle the samples was distributed and available for all participants’ caregivers or assistants. To collect saliva samples in adults with an intellectual disability with the help of a parent or a caregiver was considered feasible^17^. The home samples of saliva were collected equally for all participants whether belonging to the intervention or control condition. The samples were collected in the morning and evening on the day, one week before the first session; on the days of the 1st, 6th and 12th sessions; and on the day, one week after the last session. The morning saliva was collected in bed after awakening and before the participants brushed their teeth or had breakfast, typically between 7:00 and 8:30 a.m. (Md = 8:00 a.m.). The evening saliva was collected at least one hour after dinner or other meal and before brushing their teeth, typically between 8:00 and 9:00 p.m. (Md = 8:17 p.m.). The saliva samples were immediately frozen at the participants home (− 18 °C) and later, in connection with post intervention measurements, transported in a freezer bag (0 °C) between one and three hours to the laboratory where they were stored in an ultra-low temperature freezer (− 80 °C).

A similar sampling equipment and sampling procedure as used at the participants’ home, was used at the pool in connection with the intervention, i.e., the swab was placed in the participant’s mouth for 60–90 s and then put into the centrifugation tube and marked with participants’ ID, date, and time. The intervention samples were collected at sessions 1, 6 and 12, right before entering (baseline) and leaving the pool, respectively. The saliva collected at the end thus reflect the first part of the SWAN sessions. Samples collected at the pool before and after the SWAN sessions were put in a freezer bag (0 °C) and between one and four hours later, put in an ultra-low temperature freezer (− 80 °C) at the laboratory.

#### Salivary cortisol analysis

All saliva samples were assayed for cortisol in duplicate using a highly sensitive enzyme immunoassay (Salimetrics, State College, PA). The test used 25 µL of saliva per determination. The lower limit of sensitivity was 0.2 nmol/L. The average intra-assay coefficient of variation was 5% and the average inter-assay coefficient of variation was 7%. A random selection of 9% of the samples was analysed twice in order to determine reliability of the assay. The correlation was r = 0.95 (p < 0.0001).

#### Assistants’ assessments

The two assistants that accompanied the participants assessed the participants’ degree of stress in agreement on a 5-point Likert scale, from 0 = no stress at all, to 4 = very much. We did not explain any particular signs to look for except for signs of stress in general. Instead we asked the assistants, all of whom knew the participant well, to be attentive to the participants specific signs (sound, facial expressions, and motor behaviour) indicating stress. The assessment was completed thrice at each session: before the session started when the participant was in the locker room, during the water dance session when the participant was in the pool, and finally after the session when the participant was back in the locker room.

### Statistical analysis

All analyses were conducted using the IBM Statistical Package for the Social Sciences for Windows, version 25.0 (IBM Corporation, Armonk, NY, USA). The raw cortisol values were non-normal according to the Shapiro–Wilk test (p < 0.05) and consequently the cortisol data were natural logarithm transformed^31^. Thus, all analysis was performed on ln-transformed data; however, untransformed data were used in figures and tables for clarity. The ln-transformed values were approximately normal distributed with a skewness of − 0.006 with a standard error of 0.088 and a kurtosis of − 0.394 with a standard error of 0.176. No outlier was identified. Linear mixed model (LMM) analysis with restricted maximum likelihood was chosen because it uses all the available information in data in a repeated-measures design and is robust in handling missing data^32^. A separate linear mixed model was built for the two outcome variables, assistants’ ratings, and salivary cortisol. All models included random intercepts, and unstructured components analysis was used to account for within-subject correlation over time. Significance levels were set at 0.05 (two-sided).

### Ethical considerations

The study was performed in accordance with the Declaration of Helsinki. Regarding the profound disability of the participants, all of them had a legal guardian who gave written informed consent after reading information about the study and receiving information from the researchers via telephone or in person. The risk of discomfort or harm for the participants was not considered higher than in a regular course of treatment. Nevertheless, if observed, the participants’ assistants and legal guardians were told to be vigilant on signals of discomfort or harms and to immediately notify the SWAN leader or the responsible researcher. No harm or discomfort was reported, except discomfort from the person who withdrew participation after the first session. The study was approved by the Regional Ethical Review Board in Uppsala, Sweden (dnr: 2018/070).

## Results

### Adherence

The participants attended 3–12 (M = 9.3) of the 12 SWAN sessions. Twenty-one participants (59%) attended ten sessions or more and only two participants (6%) attended less than half of the sessions. Their low attendance was explained by arising health issues not related to the intervention. There was no significant correlation between adherence and assistants’ ratings of stress or salivary cortisol.

### Salivary cortisol

Given 26 saliva samples per participant there were a planned total of 884 salivary cortisol samples. Of them, 110 saliva samples (12.4%) were missing for different reasons. The most common reason was samples missed at the participants’ home (55 saliva samples), followed by participant not attending a SWAN session (38 saliva samples), and problems to analyse the saliva samples, such as too little saliva (17 saliva samples).

#### Diurnal cortisol variation

**Figure 2:**
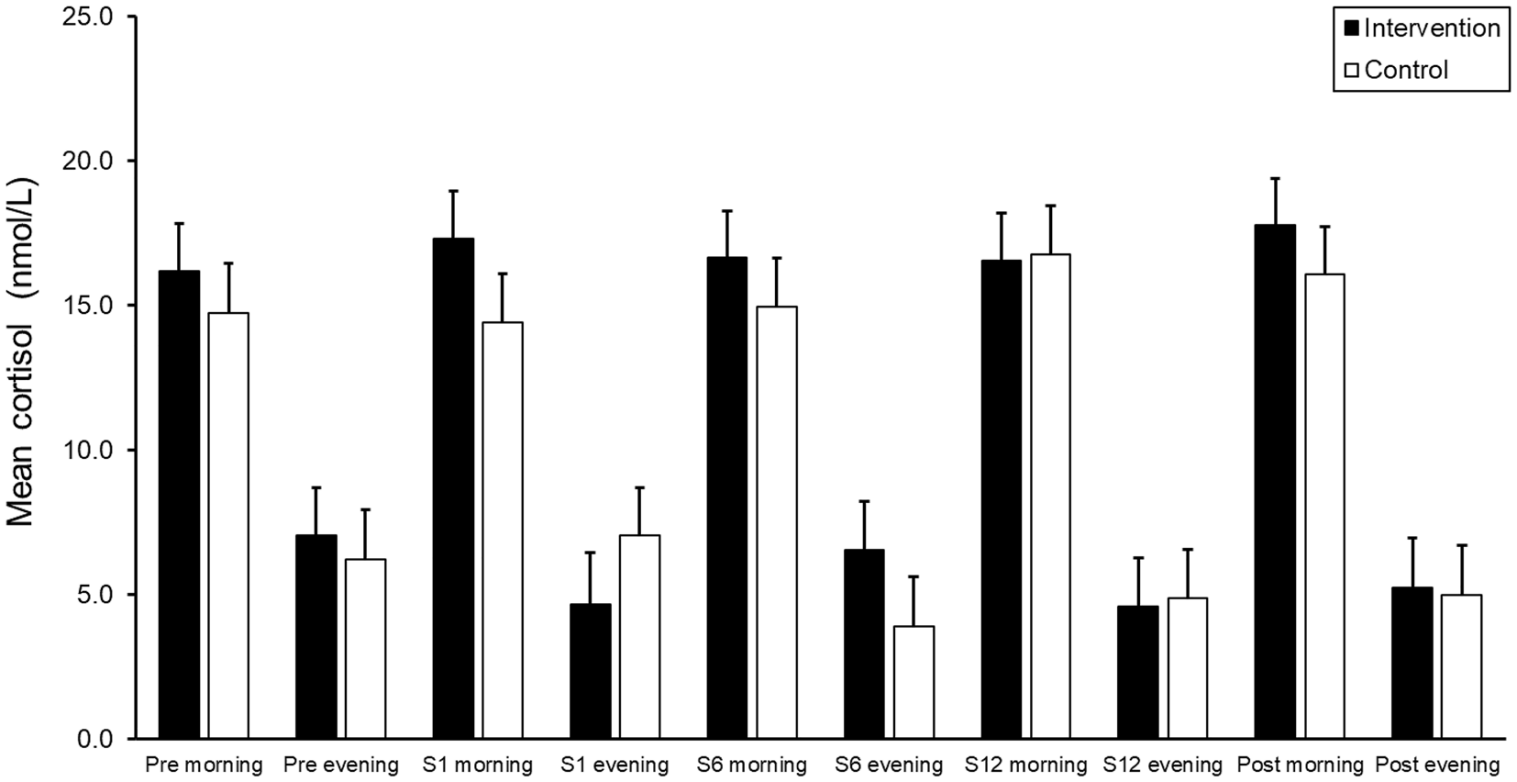
Salivary cortisol level in the morning and evening at pre intervention; sessions (s) 1, 6, and 12; and post-intervention, for the intervention and control conditions. Error bars represent 1 standard error.

Cortisol followed the expected diurnal pattern of the typical population with high levels in the morning and low levels at evening. At baseline, morning and evening, salivary cortisol had a mean of 14.82 nmol/L (range 1.57–43.90; SD = 8.40) and 4.39 nmol/L (range 0.83–23.70; SD = 4.49), respectively. As shown in Fig. 2 and supported by the LMM results, the cortisol levels were higher in the morning and lower in the evening (*F*_*(1,557)*_ = 489.19, *p* < 0.001), whereas the morning and evening differences did not change significantly across the study period i.e., there was no significant difference in morning and evening cortisol between the intervention and control condition.

#### Daily measures by session

**Figure 3:**
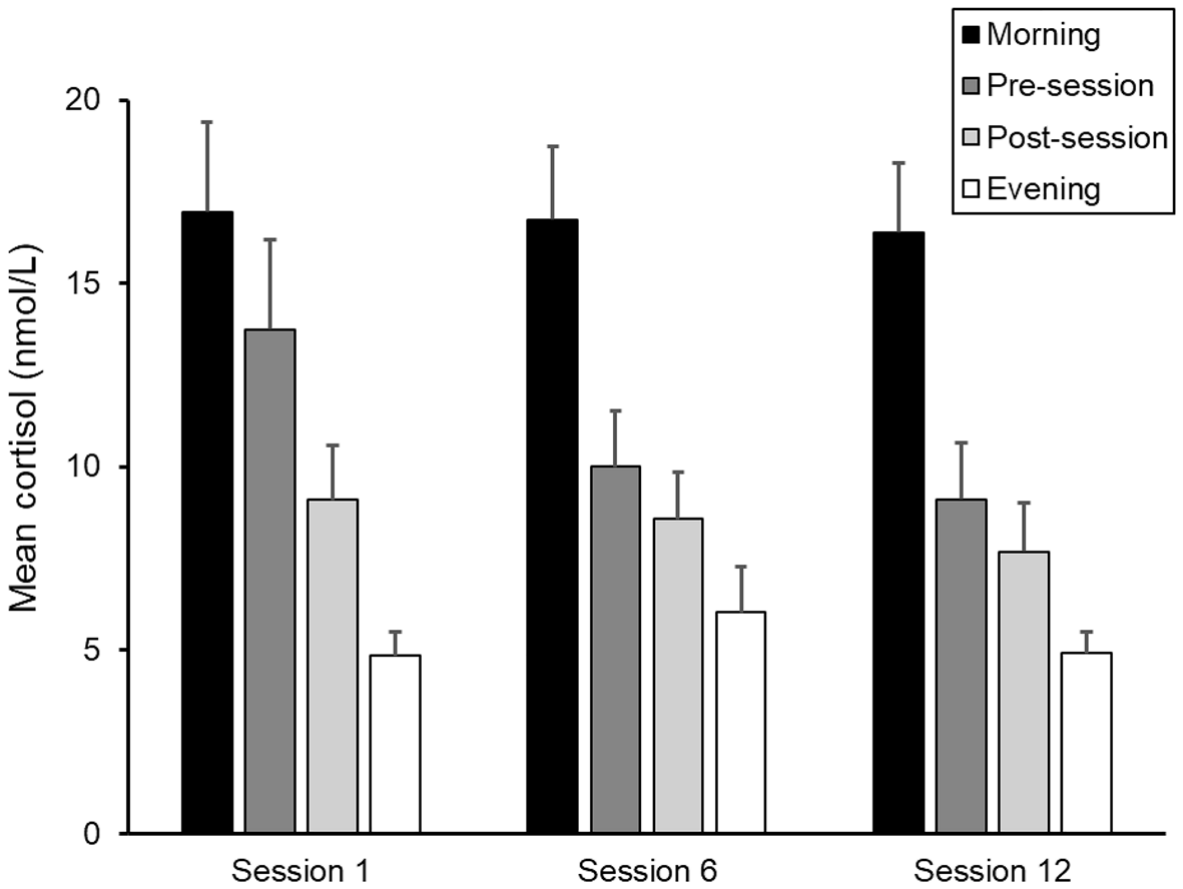
Salivary cortisol level in the morning, before session, after session, and in the evening, at sessions 1, 6 and 12 in the intervention group. Error bars represent 1 standard error.

As shown in Fig. 3, the cortisol levels decreased during the day as expected and this decrease was supported by a significant time of day effect of the LMM analysis (*F*_*(3,30)*_ = 36.95, *p* < 0.001). There were no significant main effect of session or time of day by session interaction effect.

#### Before and after session, by sessions

**Figure 4:**
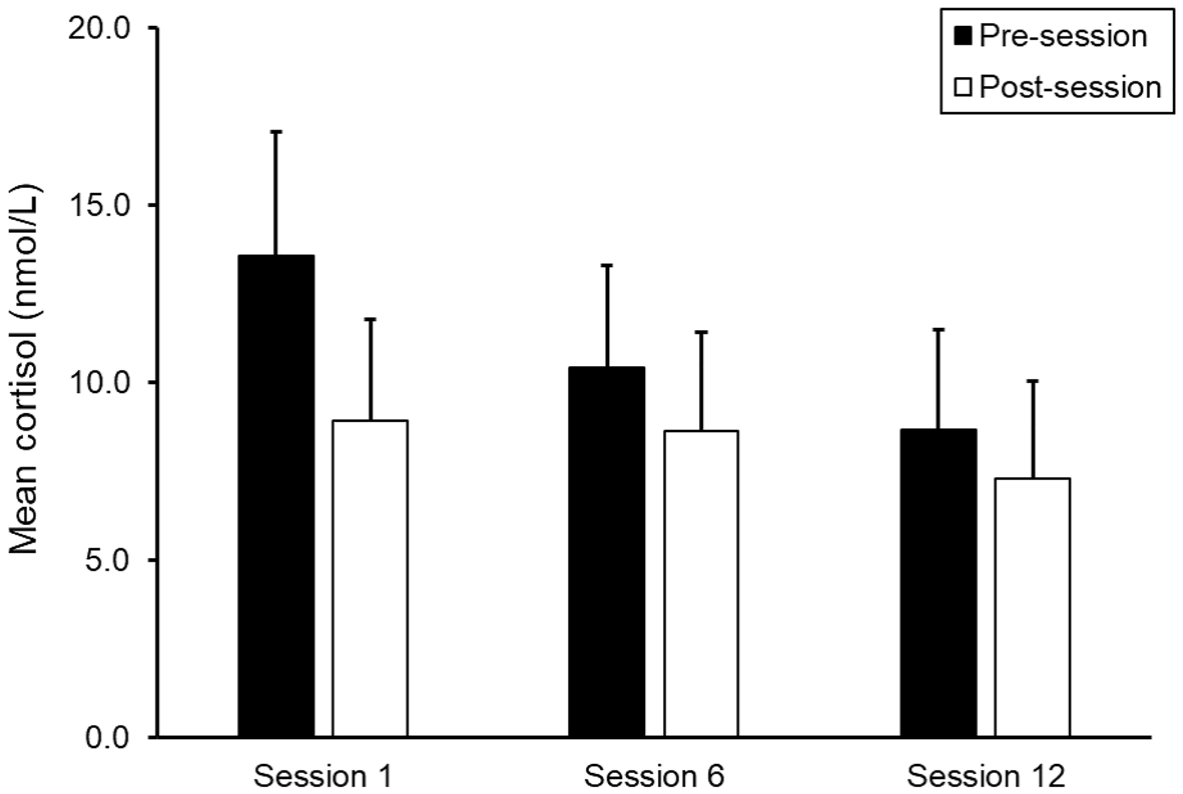
Mean salivary cortisol before and after SWAN sessions 1, 6 and 12 in the intervention group. Error bars represent 1 standard error.

As shown in Fig. 4, the salivary cortisol levels decreased within the sessions and were lower at post-sessions than at pre-sessions. The LMM revealed a significant pre-post session effect (*F*_*(1,32)*_ = 18.14, *p* < 0.001), but only approached a significant session effect (*F*_*(2,27)*_ = 2.70, *p* = 0.086) and a pre-post session by session interaction effect (*F*_*(2,26)*_ = 3,30, p = 0.053). Follow-up analyses of the interaction effect showed that the pre-post session cortisol level decrease at sessions 1 and 12 were significant (*F*_*(1,28)*_ = 18.00, *p* < 0.001 and *F*_*(1,27)*_ = 6.63, *p* = 0.016, respectively) whereas the decrease at session 6 was not (*p* = 0.12). In addition, the pre-session cortisol level decreased significantly across the sessions (*F*_*(2,23)*_ = 3.584, *p* = 0.044) whereas the post-session cortisol levels were fairly stable across sessions (*p* = 0.231).

### Assistants’ ratings

Given 36 ratings per participant (i.e., three ratings of stress per session) there were a planned total of 1224 ratings. Of them, 934 (76.3%) were complete, 273 ratings (22.3%) were missing because the participants did not attend a SWAN session, and 17 ratings (1.4%) were missing because the assistant forgot to complete them or were distracted.

**Figure 5:**
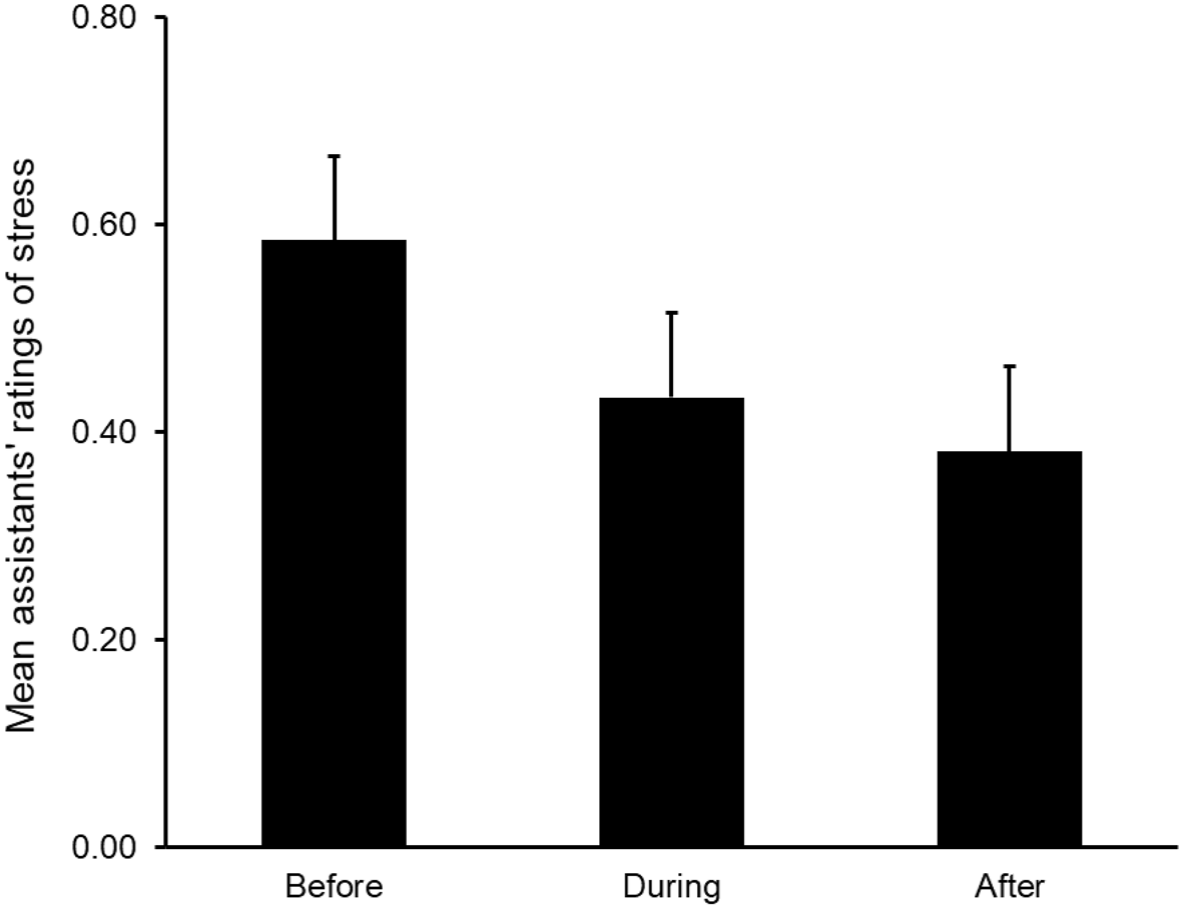
Mean assistants’ ratings of participant’s stress before, during and after the SWAN sessions. Error bars represent 1 standard error.

The LMM analysis of the assistants’ ratings of participants’ stress showed a significant within-session effect (*F*_*(2, 868)*_ = 7.13, *p* < 0.001) but no significant between-session effect or interaction effect. As seen in Fig. 5 and supported by follow up tests, the assistants’ ratings of the participants’ stress were significantly lower for the ratings made during the sessions compared with the ratings made before the sessions (*F*_*(1,570)*_ = 7.13, *p* = 0.008) and those made after the sessions (*F*_*(1,569)*_ = 12.29, *p* < 0.001). There were no significant differences between the ratings made during the session and the ratings made after the sessions.

Since both assistants’ assessment of stress and salivary cortisol decreased after sessions, we correlated the change in these two variables, which gave a small and insignificant positive correlation (*r* = 0.26, *p* = 0.170).

## Discussion

The results demonstrated that the salivary cortisol levels and the assistants’ assessment of participants’ stress decreased in response to the SWAN sessions. In addition, the pre-versus post-session difference in cortisol levels decreased across sessions. This was mainly due to the fact that the pre-session cortisol levels were highest at the first session and decreased during the intervention period, while the post-session cortisol levels were fairly constant across sessions. One possible explanation to this effect is that participants were more excited at the first SWAN session. As they became more familiar with the intervention at sessions 6 and further at session 12, their expectations decreased, as so did their cortisol levels. This effect was, however, not observed in the assistants’ assessment of stress; instead, the decrease in assessed stress after SWAN sessions was stable across the intervention period. It is worth noting that the decrease in cortisol levels from pre- to post-session could be a result of the natural cortisol decline during the day. However, cortisol levels normally decrease steadily during the day and the observed decrease following a 40-min session indicate a steeper decline than expected. In addition there may be some potential confounding factors affecting salivary cortisol levels, such as lifestyle factors, daily rhythm, depression, medication to mention a few. However, all participants completed the intervention and any individual differences would thus be cancelled out. Although, we cannot exclude the existence of more complex relationships between these confounders and the effects of the SWAN intervention.

Although both salivary cortisol and assistants’ assessment of stress decreased as a result of the SWAN intervention, there was no correlation between these variables. This is in line with previous research demonstrating extremely low correlation between salivary cortisol and subjective stress^33–35^. In our study this may indicate that salivary cortisol and assistants’ assessments are based on different mechanisms. The assistants must rely on nonverbal cues of the patient. These cues may not be related to the patient’s cortisol level. Instead, it could be asynchronised reactions due to a relatively slow secretion of cortisol into the bloodstream and saliva. Thus, the effects of SWAN on the participant's salivary cortisol levels may be present after the end of the session when they have returned home. Hence, future studies on the temporal relationship between proxy ratings of subjective stress and salivary cortisol is needed in people with PIMD.

Previous studies propose that people with PIMD may have higher stress levels^4^ or be more prone to experiencing stress in novel situations^6,7^. These assumptions were not supported by the present study. Instead, it demonstrated that people with PIMD participating in an aquatic activity show reduced stress levels. This is an important clinical finding. Since people with PIMD cannot communicate their feelings verbally, caregivers may be left in uncertainty to whether hydrotherapy activities are pleasant or unpleasant.

Despite previous research suggesting divergent diurnal cortisol variation for individuals with neurological conditions, the participants in the present study showed expected diurnal cortisol pattern in line with studies on typically developing individuals^9,36^. Considering the lack of studies on cortisol levels in adults with PIMD, the result is an important contribution to the knowledge of diurnal cortisol levels of this group of individuals.

The present study has some limitations that are important to consider when interpreting the results. First, the prevalence of PIMD is low; hence, we used a multicentre design to increase the population basis of the sample. Despite that, we were only able to recruit 36 out of the 40 participants indicated by the initial power analysis. Consequently, the power of the study was not optimal and a larger sample size might have revealed further significant effects of the SWAN. However, it cannot be ruled out that the relatively small sample size and the number of missing values and dropouts may affect the estimated covariance structure. Thus, the results should be interpreted with caution until they are replicated. Moreover, the morning and evening saliva samples collected at participants’ homes were exposed to thawing in transit to the laboratory, which might have affected the results of the salivary cortisol assay. However, studies show that cortisol in saliva is stable to freeze thaw sequences^9,37^. In addition, since all participants were included in both the intervention and the control conditions, a participant’s morning and evening salivary samples during the intervention and the control condition would have been exposed to the same freeze thaw cycle in transit to the laboratory. Thus, even if thawing had an effect, it would have similarly affected the salivary samples. Furthermore, the saliva cortisol peaks 25–30 min after exposure to a stressor. Therefore, the sample collected after the end of the SWAN session may reflect the potential stress level during the first part of the session. In addition, although the lab personnel analysing the salivary cortisol samples were blinded, it was not possible to blind the assistants rating the perceived stress of the participants since they had to be present when the participant was receiving the intervention. The two assistants made their assessment in agreement. One of the assistants was at the pool side and the other in the water with the participant. In that way they may have complement each other's perceptions of the participant’s stress. Although, this setup does not allow for assessment of interrater reliability, by using both salivary cortisol and ratings of stress as independent assessments on the same construct, we tried to reduce this risk of assessment bias.

This is the first attempt to evaluate the effect of SWAN on reducing stress in individuals with PIMD. As demonstrated above, evaluating any method on groups of people with rare and severe disorders is a challenge. Hence, the present study needs to be replicated in another and preferably larger sample of people with PIMD as well as in other settings and contexts. In addition, studies comparing SWAN to other interventions directed to reduce stress, such as massage, relaxation programs, etc., is warranted. The evaluation of SWAN was made on the intervention as a whole, thus we do not know the effect of the music, the movements in warm water, the social interactions and the performance in a group versus individually. Therefore, future studies may focus on determining the impact on the constituent ingredients of SWAN.

In conclusion, the present study demonstrates that the SWAN reduces salivary cortisol levels and proxy rated stress in adults with PIMD, which justifies that SWAN, on short-term, could be considered when choosing interventions to reduce stress in adults with PIMD. In addition, the observation that the diurnal salivary cortisol variation patterns were consistent with that of adults without disability, contributes to the limited evidence regarding cortisol levels among adults with PIMD, in this field.

## Data Availability

The datasets generated during the current study are not publicly available due to legal and ethical restraints. Study data are available from the corresponding author on reasonable request.
